# Dietary Intervention through Flipped Learning as a Techno Pedagogy for the Promotion of Healthy Eating in Secondary Education

**DOI:** 10.3390/ijerph17093007

**Published:** 2020-04-26

**Authors:** Juan Antonio López Núñez, Jesús López-Belmonte, Antonio-José Moreno-Guerrero, José Antonio Marín-Marín

**Affiliations:** Department of Didactics and School Organization, University of Granada, 18071 Granada, Spain; juanlope@ugr.es (J.A.L.N.); ajmoreno@ugr.es (A.-J.M.-G.); jmarin@ugr.es (J.A.M.-M.)

**Keywords:** dietetic, education research, educational innovation, educational technology, teaching, flipped learning, methodological contrast

## Abstract

Technological progress in the educational field has led to the application of active and innovative teaching methods, such as flipped learning, including in the field of dietary education. This is considered a mixed formative approach that combines face-to-face and outside the classroom education. The objective of this research was to analyze the effectiveness of flipped learning methodology on a traditional training practice in dietary training, both in the sixth grade of primary education and in the fourth level of secondary education. A quasi-experimental design was adopted with two experimental groups, two control groups and only posttest. The final sample was composed of 115 students divided into four groups, two of each educational stage mentioned. A didactic unit consisting of six sessions in all groups was applied. Two different training methodologies were followed according to the nature of the group (control-traditional; experimental-flipped learning). The results reveal that flipped learning is effective both in primary education and in secondary education, being more influential in student development in this last stage. It is concluded that the flipped learning approach has meant an improvement of the academic indicators evaluated after a diet education program.

## 1. Introduction

The advancement of information and communication technologies (ICT) is generating new trends and ways of acting in people’s daily lives. This fact does not go unnoticed in the various social sectors, specifically in education [1], where a process of constant digitalization is taking place [2]. Technological innovations in teaching are generating new training processes, thus promoting innovative educational praxis [3]. Training actions linked to the use of ICT [4], also called techno-pedagogical [5], promote a series of potentials such as the exchange of roles between educational agents [6], the emergence of new teaching methods [7], the use of new resources and educational materials [8], training development anywhere and at any time [9], and access to a large amount of information [10].

All this generates new scenarios in teaching and learning processes [11], which cause improvements in attitudinal, aptitude, and performance aspects in the students themselves [12]. Therefore, we are facing a process of pedagogical renewal [13]. An example of technopedagogy is flipped learning (FL) teaching method [14].

### 1.1. The Use of Flipped Learning in the Educational Field

FL can be defined as a teaching-learning method or process that combines the face-to-face plane with the outside the classroom [15]. This techno-pedagogical praxis is booming in the academic world [16], since it is being used at all educational levels [17], allowing for structuring of academic experiences to achieve a greater learning result [18] due to the effectiveness, the practicality, and the dynamism that it generates in the instructive processes [19]. This method is increasingly used by teachers, who are getting better results in their students with this innovative methodology than with the use of a traditional approach [20].

The configuration of FL tries to turn the traditional teaching acts [21,22], allocating the outside the classroom period to acquire, assimilate, and settle the theoretical contents proposed for the subject [23] and dedicating the face-to-face period to solving problems and developing practical actions [24] through direct and continuous interaction of the teacher with the students and of the students themselves [25] (Table 1). Although this does not ensure a greater connection and awareness by parents of their children’s teaching and learning processes, it can lead to an improvement in the training process, obtaining advantages and reporting potentialities at the academic level [26].

This teaching method requires an effort by the teacher [27], since they must generate educational content, sometimes under audiovisual support [5], host them on a digital platform with easy access for students [28], and propose work dynamics for the face-to-face period [29], promoting high quality teaching and learning activities in the classroom based on the autonomous study of the students. Students also make an effort to take an active formative action and are responsible for their own learning [17]. However, the research results focus on specific contexts, the evidence for which must be treated with caution [30].

The use of FL can promote and generate a range of advantages at the academic level, such as an increase in the interaction between students [31] and between the teacher and the student [23], an increase in motivation [32], increased participation [33], improved attitude towards the training process [34], greater commitment to the task [35], adaptation of the pedagogical act to the individual characteristics of the students [36], greater autonomy on the part of the student [37], increased socialization between educational agents [38], and increased academic performance [39]. All this can generate improvements in student ratings [40], a positive effect on learning outcomes [41], and greater assimilation of the curricular elements proposed for the subject [42].

### 1.2. Dietary Habits as a Factor of Health and Development in School Children

Nutrition has a high impact on the health status of people [43], since a diet based on its quality can prevent diseases [44] or cause them [45]. In this sense, the fact of being overweight or obese can generate serious public health problems [46], affecting the increase in the mortality rate [47]. In the educational field, the assimilation of good eating habits in students [48] acquires great relevance, given that the actions developed through transversal treatment or nutritional education programs promote adequate dietary guidelines [49,50] while avoiding bad praxis, among which is the intake of sugars and other substances harmful to health [51,52], especially in those people who have various diseases, such as diabetes or cardiovascular problems [53,54].

Currently, educational actions related to nutrition are increasing through the use of innovative methodologies such as distance education [55] or emerging technologies such as augmented reality [56], which are turning out to be effective methods both in learning and in changing dietary habits [57]. In addition, pedagogical actions in which healthy diets are promoted and associated with moderate physical activity lead to improvements in the organism at the arterial level, in the body mass index, in the decrease of the fat index, and in the decrease of cholesterol [58]; it is even determined that it has a positive impact on students’ academic performance [59].

### 1.3. Justification and Objectives

Recent studies support the use of active methodologies such as FL to carry out a training action where students are the main protagonist and builder of their own knowledge through the use of educational technology that is constantly evolving as a consequence of the incidence of an increasingly digital society [11,60].

To verify the different findings postulated by experts in this field of knowledge, this research is presented—with an exploratory nature—on dietary education, justified in the absence of studies certifying the effectiveness of a methodological contrast (FL-traditional) to training level in two different educational stages (primary and secondary education). This experimentation allows us to offer new findings to the scientific community about the state of the matter. In addition, this work reduces the gap in this field of knowledge found in the impact literature, establishing a starting point for future studies.

In addition, there are few studies that analyze active teaching methods for the training of dietetic education, focusing mainly on the collaborative method [61], thus this study aims to provide a pedagogical proposal for training in dietetic education from an innovative perspective in a branch of education that is reaching great relevance today due to eating disorders that occur in today’s society [62].

After analyzing various recent impact studies that carried out training practices and experiences using FL in different subjects and educational levels, it was verified that they all follow the same methodological pattern, that is, the application of this approach mostly uses a similar instructional procedure [63,64,65,66,67,68]. The processes are synthesized in the previous visualization of videos outside the school environment, followed by the carrying out of practical activities in the educational center, then ending with a reinforcement again in digital media to solve doubts that have arisen and to visualize new audiovisual content of the next sessions formative [21,22,23,24,25]. Therefore, the literature reflects how FL implementation process does not differ according to subjects, content, or educational levels. The scientific and teaching community follows the steps, the principles, and the methodological guidelines established by the forerunners of this innovative teaching approach, Jonathan Bergmann and Aaron Sams [21], in order to carry out an optimal teaching and learning process adapted to new times through which education runs and, above all, respecting the raison d’être of this approach. However, FL approach, when presenting some intrinsic formative peculiarities by nature, as previously mentioned, does change with respect to other instructional methods, since each one has its characteristics, tools, and methodological processes that make them different and unique [60]. The present study, despite not having previous literature in which to lean within the field of dietetic education due to the absence of works that have experienced FL in contents alluding to said field of knowledge, aims to explore the potentialities of this focus on dietary education versus traditional teaching, as it has already been revealing in other knowledge and areas of knowledge.

The purpose of this research is to continue the path initiated by other studies conducted on FL in different formative contexts [69,70,71,72,73,74], where it is demonstrated that the potential of FL is better in secondary education than in primary education [5,14,17,75]. The staging of FL helps to carry out training practices typical of an era where technology and methodologies based on its use acquire a relevant value in the educational field [76]. In this study, there is a contrast between an innovative methodology such as FL with a traditional exhibition methodology based on the transmission of content by the teacher and orally, without the use of digital resources [77].

The objectives of this research focused on: (1) checking the effectiveness of FL on a traditional methodology in sixth grade primary education and fourth year of secondary education; (2) determining the course that obtained the best results in the experimentation. The specific objectives that conducted the investigation were the following:

1. To specify the level of motivation of the students.
2. To find out the level of interaction.
3. To know the level of autonomy of the students.
4. To discover the level of collaboration of students.
5. To determine the level of deepening of the didactic contents.
6. To find out the level of problem solving.
7. To discover the level of class time.
8. To determine the influence in the qualifications.

## 2. Materials and Methods 

### 2.1. Research Design and Data Analysis

The study was carried out through a quasi-experimental design of a descriptive and correlational type based on a quantitative methodology of statistical treatment of the data, according to the specialists in this field of analysis [78,79]. Likewise, the investigative structure of recent studies of the impact literature was followed in order to follow a validated research model [5,14,80].

The design used required the establishment of two types of groups (control = CG; experimental = EG). The difference between groups was established at the formative level. The CG followed a traditional instructional action. The EG developed the training through FL approach. This group configuration established as an independent variable the type of training methodology and as a dependent variable the effectiveness obtained in the academic items used in the experimentation.

All information collected was managed with the Statistical Package for the Social Sciences (SPSS) v25 program (IBM Corp., Armonk, NY, USA). To extract the results, several statistics were used, such as mean (M) and standard deviation (SD). The distribution trend was determined with skewness (S_kw_) and kurtosis (K_me_). The comparison of the means between CG-EG was carried out with the t-Student test (t_n1 + n2-2_). The effect size was obtained with Cohen’s d and biserial correlation (r_xy_). A *p* < 0.05 was established as a level of statistical significance.d

### 2.2. Participants

The experimentation was carried out with a sample of 115 students from an educational center in Spain. For this, an intentional non-probabilistic sampling technique was used. Regarding the volume of participation, the literature states that the number of subjects in this type of study does not affect their performance and does not obtain significant results [81,82].

Of the students chosen, 57.39% were boys and the rest girls with an average age of 14 years (SD = 2.91). Students were enrolled in sixth grade of primary education and fourth year of secondary education. These courses were chosen because they are the last level of each educational stage. This favors the comparison between stages because the students have already reached the last year and have worked on the different skills of each stage in its entirety. These aspects taken into account justify the choice of the chosen courses and favor the generalization of the results achieved.

With the sample reached, four groups were established. Two control groups (primary education = CGP_1_; secondary education = CGP_2_) and two experimental (primary education = EGP_1_; secondary education = EGP_2_). As indicated in Table 2, the applied treatment (innovative FL methodology) was carried out in a probabilistic way in the experimental groups, and only a single final measurement was made in each of the groups.

### 2.3. Instrument

The data were collected by an ad hoc questionnaire. This tool was made according to different instruments found in the expert literature on FL [5,14,78,81,82]. At a structural level, the questionnaire is articulated in nine dimensions (socio-educational, motivation, interactions, autonomy, collaboration, content deepening, problem solving, class time, and ratings) with a total of 35 items that follow a response format in Likert scale (from 1 = none to 4 = completely). In addition, the qualifications collected by the teacher were taken into account.

The validity of the questionnaire was achieved in two processes, both qualitatively and quantitatively. The Delphi method was the procedure used for qualitative validity. Eight university doctors’ experts in active and emerging methodologies were selected. These professionals analyzed the format, the structure of the questionnaire, and the various items. The assessment was positive (M = 4.64; SD = 0.37; min = 1; max = 6). The observations and the proposals for improvement were focused on the reduction and the grouping of some issues and on the modification of the lexical level of certain items, with the intention of improving the understanding of the issues. All expert recommendations were made to optimize the instrument and reduce bias due to participants’ misunderstanding.

For quantitative validity, the Kappa of Fleiss and W of Kendall tests were used to analyze the judgments offered by the specialists. These statistics revealed an adequate level of concordance and relevance of the feedback delivered (K = 0.84; W = 0.86).

An exploratory factor analysis by the principal component’s method was the procedure used for the quantitative validation of the questionnaire. For this purpose, several tests were carried out, such as the Bartlett’s test of sphericity, which determined dependence between the variables (2613.28; *p* < 0.001) and the Kaiser-Meyer-Olkin test, which revealed a relevant adequacy of the sample (KMO = 0.87).

To obtain the reliability of the questionnaire, several statistical tests were used, such as Cronbach’s alpha (α) (0.86), compound reliability (0.84), and mean variance extracted (0.81), which reflected adequate internal consistency indices in the items presented.

### 2.4. Study Dimensions

The dimensions analyzed in this study were taken from other studies reported in the impact literature on the state of the matter that analyzed the incidence of FL in other subjects, social contexts, and educational levels. The dimensions are described below to facilitate the interpretation of the results obtained. In addition, for greater scientific rigor, the choice of each dimension is supported by previous studies, where the adequacy and the relevance in the use of such dimensions is verified. 

Socio-educational encompasses aspects related to gender, age, city, nationality, religion, course, learning difficulties, training methodology, and use of digital resources [14,60,73,78,83,84,85,86]. Motivation refers to the degree of motivation of the students during the learning process [14,60,73,78,83,84,85,86]. Interactions groups the type of interaction possible in learning actions such as the interaction between the teacher and the students, between the students and the didactic contents, and, finally, between the students [14,63,78,83,84,85]. Autonomy determines the degree of autonomy reflected by the students in carrying out the various training activities, both teaching and learning [14,60,67,78,83,84,85,86]. Collaboration refers to the degree of teamwork achieved by students in the instructional process [14,73,78,83,84,85]. Content deepening reflects the degree of projection (greater or lesser dedication) of teachers in the contents according to the training methodology used [14,73,78,83,84,85]. Problem solving reveals the degree of competence of the students to attend and solve the contingencies originated or proposed during the formative action. Class time refers to the temporary availability to impart, work, and reinforce the contents by the educational agents [14,73,78,83,84,85]. Ratings refers to the grades obtained by students in the assessment test performed to measure assimilated knowledge. These dimensions, which measure the level of knowledge acquisition by the students, were carried out by means of the questionnaire, in which they were asked: what is your average mark in general? what is your general average in the subject of physical education? and what has been the mark you have obtained in the subject of physical education after the development of the experience? The relevance in the use of this dimension is justified by previous studies that reflect its proper use to measure the learning results achieved by students [14,73,78,83,84,85]. The teacher ratings dimension includes the student’s ratings according to the teachers who taught the subject. In both cases, the same assessment techniques and instruments were used. In other words, the written test was used, which was worth 60% of the final rating of the subject, along with direct observation, with a weighting of 20% of the final rating, and the portfolio, with a weighting of 20% [86].

### 2.5. Procedure

To carry out the experimentation, several processes had to be carried out. At first, the educational center was selected—a school in southern Spain that contains several educational levels. Afterwards, a meeting was held with the representatives to explain the purpose of the study and obtain permission to access the sample. Next, the participants were chosen intentionally, and the analysis groups were set up. By having two student groups for each educational level, the allocation of control group and experimental group occurred randomly. Then, the training phase began in which a didactic unit of dietary education was developed in the subject of physical education within the health content block. The teaching unit was composed of six sessions, and the following content was taught: (a) healthy life; (b) feeding habits; (c) dietary guidelines; (d) harmful consequences for health.

Methodologically, the teaching unit—according to the group of students—was carried out differently.

In the control group, the teacher developed the different sessions in a traditional way. No digital tools were used in this group. The teacher became the only source of knowledge transmission. The students assumed a passive role, their only task being to listen to the explanations and carry out the training activities in the classroom. These activities consisted of the realization of files with activities related to the contents taught orally by the teacher. The activities were carried out individually and consisted of answering various issues related to the subject in writing. All the formative action, both the teaching and the learning of the contents, occurred physically in the classroom. No action was taken outside the school space. The teacher spent a short time in the classroom to perform the activities. Students finished home activities not completed in class without any teacher support in the space outside the classroom.

The experimental group performed a learning process through FL. The teacher generated audiovisual teaching material. These resources were stored on a content platform so that students could view them anywhere outside the school environment and before the classroom session. The content delivery process occurred digitally and autonomously by the students. This allowed other activities focused on research, teamwork, and problem solving to be carried out in the classroom. This allowed the development of a variety of training activities and a longer class time for its realization, because the explanations of the contents were transferred to a previous digital space. Therefore, the students became active agents in the construction of knowledge. The students achieved greater prominence during the learning process by having to visualize the audiovisual material in other learning spaces outside the school and perform different training activities on the contents displayed in different formats (answer questionnaires, find information on the subject, solve issues and problems raised by the teacher collaboratively with other students). The audiovisual material was always available so that the students could view it at any time to answer their questions or reinforce the contents. For all this, the guidance of the teacher during the activities carried out in the classroom was essential.

The last phase consisted of applying the questionnaire and analyzing the data collected at the statistical level in order to respond to the objectives formulated in the research in addition to increasing the literature on the application of emerging methodologies—in this case FL, for the delivery of content related to healthy dietary habits.

## 3. Results

According to the data obtained in the descriptive analysis, specifically in the group of primary education students, the means presented by the CG were below two points in all the variables analyzed, except in motivation, student–student, and collaboration, which were slightly above. In the EG, the means reached were above 2.5 points in all variables, except for student–content, student–student, deepening, and resolution, which were slightly below. Differences in ratings between students and teachers varied but were minimal. Ratings were higher according to the teachers. The values in the variables of the control group and the experimental group, taking into account what was marked by [87], offered a normal distribution, since they were between −1.96 and +1.96. The standard deviation showed a distribution of response matched by the participants in all the variables in both groups, except in collaboration and class time, of the CG, and deepening, resolution, and class time of the EG, where the response was more dispersed. The kurtosis shown in all the variables was platykurtic, except in ratings, where it was leptokurtic, and in student–content, where it was mesokurtic, both of the CG (Table 3).

In secondary education students, the CG averages were below two points, except in motivation, student–student, and collaboration, which were slightly above that average. In contrast, the average obtained by the EG in all the variables analyzed was located above 2.5 points. In this case, as in the past, the differences in ratings between students and teachers varied, although these differences were minimal. Ratings were higher according to the teachers. With the values of the standard deviation in mind, the answers given by the CG were more evenly matched, while those given by the EG were more dispersed. With respect to kurtosis, it was mostly platykurtic, except in teacher–student, deepening, and CG ratings, which were leptokurtic (Table 4).

**Figure 1:**
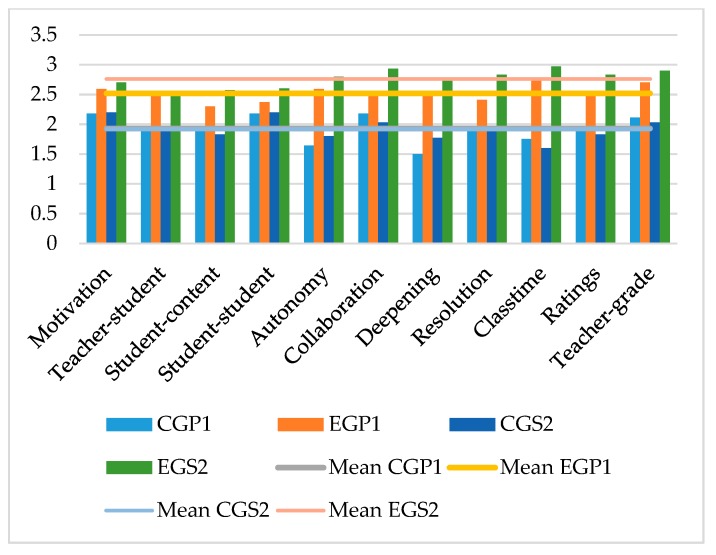
Comparison between control group primary education (CGP_1_), control group secondary education (CGP_2_), experimental group primary education (EGP_1_), experimental group secondary education (EGP_2_).

In the comparison of the students of the primary education and secondary education stages, it was shown that the measures offered by the CGs were very even with each other, showing similar values. On the other hand, in the EG, there was a difference between both educational stages, finding a higher valuation on the part of the students of secondary education than those of primary education in the developed educational experience. The data also showed that there were differences between the values of the CG with respect to the EG, with the latter valuations being higher in all the analyzed variables (Figure 1).

To determine the value of independence of the data collected between the traditional teaching method and the teaching method developed by FL, the Student *t* statistic was used for the independent samples. According to the results obtained, there were more significant differences in the course of secondary education than in that of primary education, since in the primary education stage, it turned out to be significant in teacher–student, referring to the relationship established and maintained between the student and the teacher; in autonomy, related to the capacity to develop learning and activities in an autonomous way, thus developing the learning to learn competence; in in-depth, in which the teacher, due to the tasks carried out, made it possible to present the content presented in a more detailed way; in class time, with the feeling that the student were able to learn much more quickly than is usually the case for him/her; in grades, aimed at the student’s ability to assess his/her academic development; and in teacher–class, in which the grades established by the teacher for the student were analyzed. On the other hand, in secondary education, this was the case in all the variables analyzed, except in the student–student one; that is to say, the fact of applying the teaching method by means of FL does not suppose an improvement in the relations among the own students. These results show that the flipped learning method is more effective in secondary education students than in primary education students (Table 5).

## 4. Discussion

The influence of ICT in the new millennium has overcome all kinds of borders [1]. Technology has reached the educational field to produce change and improve and enhance learning processes [2,3,4,5,6,7,8,9,10]. FL is a product derived from the constant evolution of educational technology [11,12,13,14]. This hybrid learning method, by combining both face-to-face and outside the classroom aspects, has allowed the incentivizing and the dynamization of training activities [15]. Expert literature in this field of knowledge reflects how the application of FL reports a learning benefit made by students [19]. Recent research analyzed various academic indicators and verified how the application of FL contributes to improving the indexes of both academic (content deepening, class time, and ratings) and psychosocial variables (motivation, student–content–teacher interactions, autonomy, collaboration, and problem solving) with respect to the implementation of other methodologies, such as the traditional one of an expository nature [5,14,16,17,18,19,20,30,31,32,33,34,35,36,37,38,39,40,41,42,61,62,63,64,65,66,67,68,69,70,71,72,73,76].

The aim of this research was to understand the effectiveness of FL methodology on the traditional teaching method. This was developed in the sixth grade of primary education and in the fourth grade of secondary education. The data shown by the 115 participants allowed us to respond to the objectives set. In this study, the contrast made between an innovative training method, such as FL, and a traditional method without ICT support, such as the expository, allowed us to demonstrate the potential of FL regardless of the educational stage where it is implemented as well as other research reported from the literature in different contexts and didactic contents [14,17,18,78,84].

In a more concrete way, the use of FL in the educational field, as was obtained in this study, leads to improvements in motivation [33,34], in the interactions between educational agents and content [23,31], in the autonomy achieved by students [35,37], in the collaboration for the development of the training activities [33,38], in the deepening of the contents [73], in the effective resolution to the problems posed by the teacher in the learning spaces [83], in the use of a longer class time [85], and in the ratings achieved by the students in the evaluations carried out, which are linked to performance and learning outcomes [39,40,41,42].

It should also be noted that the differences between the qualifications offered by the teachers themselves and those of the students themselves varied, although the difference was very small. In this case, teachers in both primary and secondary education had higher ratings than those considered by students themselves. With respect to the comparison made between the educational stages chosen in this experiment, it was obtained that, in the secondary education stage, specifically in the fourth year, the use of FL improved more indicators analyzed and with a higher rate of improvement than in the sixth primary education course [14,78]. Particularly, the aspects enhanced in secondary education were motivation, interactions (student–content and student–student), autonomy, collaboration among students, content deepening, problem solving, class time, ratings, and teacher-ratings. However, one aspect to note is that, in the sixth year of primary education, teacher–student interactions achieved better results.

At the statistical level, the study of the value of independence allowed us to achieve more precise results among the groups analyzed. The statistics used showed greater significant differences in the fourth year of secondary education (motivation, teacher–student, student–content, autonomy, collaboration, deepening, resolution, class time, and ratings) than in the sixth year of primary education (teacher–student, autonomy, deepening, class time, and ratings). Everything had a medium associative force, except in the motivation of EG, which was lower. The effect size was very low in the aspects analyzed, except in the autonomy of the CG, where it was slightly higher.

Evidence of significant differences between primary and secondary education groups is noteworthy. As established in the results, both in the control group and in the experimental group, it was shown that there was no significant relationship in the student–student dimension, which was in the relationship established between students during the development of the teaching and learning process [31]. This may have been due to the fact that the methodology applied did not necessarily require team or group work [24] but rather maintained a more individualized and personalized attention with the students, allowing the autonomous learning of the students to be better developed, as if it was reflected in a significant way in both groups [36]. In addition, it was shown that, in primary education, the flipped learning method was less relevant than in secondary education. This may have been due to access to technological resources, where secondary education students had more facilities than primary education students [29]. Another reason may have been the degree of maturity of students, who, in the primary education stage, required closer attention from teachers. This was not the case in secondary education, where students were more independent [27].

## 5. Conclusions

With this experimentation, continuing the path of previous research, it is concluded that FL approach implies greater advantages in teaching and learning processes of content related to dietary education than the application of traditional instructional methods where the teacher simply exposes the contents orally and grants little participation to students. In addition, it is verified that, although relevant results were obtained in both educational stages, it was in the secondary education stage where FL reached better rates. Therefore, this study reveals that the use of FL to impart content concerning healthy life, feeding habits, dietary guidelines, and harmful consequences for health is effective.

The present study allows the teachers who develop their teaching in the stages of secondary and primary education to know the effectiveness of FL method in the process of teaching and learning. This research aims to provide them with the procedure to follow in order to apply it in the classroom. Additionally, with this teaching method, we wanted to show the teachers that it is possible to carry it out in the stages of primary and secondary education. Furthermore, this research shows that teaching and learning processes developed with FL are more effective and better valued by students. That is to say, it is intended to make teachers who habitually apply the traditional method reflect on it so that they begin to use other methodologies, such as FL.

The prospect of this research focuses on the promotion of innovative methodologies for teaching and learning health-related content and adequate guidelines on food. The correct assimilation of these contents in students is fundamental for the development of a healthy life free of diseases as well as the awareness of young people towards active and healthy lifestyles. This study acquires an exploratory nuance by not finding impact research that used FL to impart content related to dietary education, in the same way as that carried out in this research. This causes a gap in the scientific literature, as there are not enough studies to build a solid base of knowledge backed by experts in the field. Therefore, the scientific community is encouraged to carry out studies on the state of the matter to continue contrasting the effectiveness of this teaching and learning methodology in dietary education in other geographical contexts and educational levels. This will contribute gradually to solving the gap found in the academic literature.

This study has several limitations. Experimentation was only developed in a specific geographical context, and only one course was taken from each educational stage. Another limitation is found in the ratings dimension that, despite being justified and supported by previous studies, can cause certain imbalances in the results, as it is a report of the children themselves. Therefore, the conclusions revealed here should be taken with caution since they cannot be generalized to the world population as a whole. To solve these limitations, as a future line of research, this study is intended to be replicated in other regions and courses of the aforementioned stages in order to establish more precise comparisons in addition to looking for other indicators used in impact studies to verify the improvement of student learning and knowledge.

## Figures and Tables

**Table 1:** Comparison between expository method and flipped learning (FL).

Periods	Expository Method	Flipped Learning
Before class	The students can read something about the educational contents to be dealt with, while the teacher prepares the theoretical presentation of the contents.	Students visualize the explanations of the contents to be worked on in class previously prepared by the teacher. The teacher generates and prepares practical activities and class dynamics.
During the class	The student listens to the theoretical explanation of the teacher, who does not use any technological resources. The teacher transmits the contents orally through the traditional exhibition. The teacher has an active attitude since it is the only source of knowledge, while the student is passive; he only receives and attends to the explanations.	The student develops dynamics and practical activities during the class. The teacher supervises, advises, or corrects the actions developed by the students. The student has an active attitude, while the teacher is passive in the learning process; its function is mainly focused on guiding, guiding, and serving students individually, according to their needs and concerns.
After class	The student’s complete homework set by the teacher, based on the theoretical explanation given at school. The teacher continues to prepare theoretical presentations.	Students reinforce what they have learned in class by putting into practice the activities developed and analyzing the theoretical videos on the contents covered. The teacher continues to prepare explanatory videos and work dynamics to develop in class.

**Table 2:** Group composition.

Group	*n*	Composition	Pretest	Treatment	Postest
1-CGP_1_	28	Natural	-	-	O_1_
2-EGP_1_	27	Natural	-	X	O_2_
3-CGS_2_	30	Natural	-	-	O_3_
4-EGS_2_	30	Natural	-	X	O_4_

Note: the treatment was assigned randomly. Two control groups (primary education = CGP_1_; secondary education = CGP_2_) and two experimental (primary education = EGP_1_; secondary education = EGP_2_).

**Table 3:** Results obtained for the variables of study in the control group (CG) and the experimental group (EG) of primary education (*n* = 55).

Variables		Likert Scale *n (%)*				Parameters			
		None	Few	Enough	Completely	M	SD	S_kw_	K_me_
**CG**	Motivation	6 (21.4)	12 (42.9)	8 (28.6)	2 (7.1)	2.18	0.905	0.269	−0.661
	Teacher–student	10 (35.7)	10 (35.7)	7 (25)	1 (3.6)	1.96	0.881	0.423	−0.765
	Student–content	9 (32.1)	13 (46.4)	5 (17.9)	1 (3.6)	1.93	0.813	0.581	−0.012
	Student–student	6 (21.4)	14 (50)	5 (17.9)	3 (10.7)	2.18	0.905	0.592	−0.129
	Autonomy	17 (60.7)	4 (14.3)	7 (25)	0 (0)	1.64	0.870	0.798	−1.21
	Collaboration	10 (35.7)	7 (25)	7 (25)	4 (14.3)	2.18	1.09	0.358	−1.18
	Deepening	19 (67.9)	4 (14.3)	5 (17.9)	0 (0)	1.50	0.793	1.19	−0.243
	Resolution	10 (35.7)	11 (39.3)	5 (17.9)	2 (7.1)	1.96	0.922	0.685	−0.247
	Class time	16 (57.1)	5 (17.9)	5 (17.9)	2 (7.1)	1.75	1.01	1.01	−0.251
	Ratings ^a^	12 (42.9)	9 (32.1)	5 (17.9)	2 (7.1)	1.89	0.956	0.441	0.858
	Teacher-ratings ^a^	10 (35.7)	9 (32.1)	5 (17.9)	4 (14.3)	2.11	1.01	0.563	−0.872
**EG**	Motivation	4 (14.8)	7 (25.9)	12 (44.4)	4 (14.8)	2.59	0.931	−291	−0.627
	Teacher–student	5 (18.5)	5 (18.5)	14 (51.9)	3 (11.1)	2.56	0.934	−480	−0.615
	Student–content	5 (18.5)	11 (40.7)	9 (33.3)	2 (7.4)	2.30	0.869	0.117	−0.552
	Student–student	4 (14.8)	13 (48.1)	6 (22.2)	4 (14.8)	2.37	0.926	0.411	−0.513
	Autonomy	3 (11.1)	11 (40.7)	7 (25.9)	6 (22.2)	2.59	0.971	0.127	−0.961
	Collaboration	4 (14.8)	8 (29.6)	12 (44.4)	3 (11.1)	2.52	0.893	−0.235	−0.567
	Deepening	7 (25.9)	6 (22.2)	8 (29.6)	6 (22.2)	2.48	1.12	−0.038	−1.34
	Resolution	6 (22.2)	9 (33.3)	7 (25.9)	5 (18.5)	2.41	1.04	0.156	−1.09
	Class time	4 (14.8)	7 (25.9)	8 (29.6)	8 (29.6)	2.74	1.05	−0.273	−1.11
	Ratings ^a^	3 (11.1)	13 (48.1)	5 (18.5)	6 (22.2)	2.52	0.975	0.347	−0.915
	Teacher-ratings ^a^	2 (7.4)	11 (40.7)	7 (25.9)	7 (25.9)	2.70	0.953	0.082	−1.07

^a^ Established grade group (none: 1–4.9; few: 5–5.9; enough: 6–8.9; completely: 9–10).

**Table 4:** Results obtained for the variables of study in the CG and the EG of secondary education (n = 60).

Variables		Likert Scale *n (%)*				Parameters			
		None	Few	Enough	Completely	M	SD	S_kw_	K_me_
**CG**	Motivation	9 (30)	10 (33.3)	7 (23.3)	4 (13.3)	2.20	1.03	0.381	−0.948
	Teacher–student	10 (33.3)	14 (46.7)	4(13.3)	2 (6.7)	1.93	0.868	0.812	0.337
	Student–content	12 (40)	12 (40)	5 (16.7)	1 (3.3)	1.83	0.834	0.715	−0.083
	Student–student	7 (23.3)	13 (43.3)	7 (23.3)	3 (10)	2.20	0.925	0.415	−0.501
	Autonomy	11 (36.7)	14 (46.7)	5 (16.7)	0 (0)	1.80	0.714	0.316	−0.911
	Collaboration	10 (33.3)	11 (36.7)	7 (23.3)	2 (6.7)	2.03	0.928	0.486	−0.623
	Deepening	13 (43.3)	12 (40)	4 (13.3)	1 (3.3)	1.77	0.817	0.876	0.340
	Resolution	12 (40)	11 (36.7)	5 (16.7)	2 (6.7)	1.90	0.923	0.773	−0.174
	Class time	16 (53.3)	10 (33.3)	4 (13.3)	0 (0)	1.60	0.724	0.794	−0.605
	Ratings ^a^	13 (43.3)	11 (36.7)	4 (13.3)	2 (6.7)	1.83	0.913	0.934	0.191
	Teacher-ratings ^a^	12 (40)	9 (30)	5 (16.7)	4 (13.3)	2.03	1.06	0.662	−0.781
**EG**	Motivation	2 (6.7)	10 (33.3)	13 (43.3)	5 (16.7)	2.70	0.837	−0.121	−0.438
	Teacher–student	6 (20)	8 (26.7)	10 (33.3)	6 (20)	2.53	1.04	−0.095	−1.11
	Student–content	2 (6.7)	13 (43.3)	11 (36.7)	4 (13.3)	2.57	0.817	0.177	−0.421
	Student–student	7 (23.3)	6 (20)	9 (30)	8 (26.7)	2.60	1.13	−0.189	−1.33
	Autonomy	2 (6.7)	11 (36.7)	8 (26.7)	9 (30)	2.80	0.961	−0.070	−1.14
	Collaboration	3 (10)	8 (26.7)	7 (23.3)	12 (40)	2.93	1.04	−0.437	−1.11
	Deepening	3 (10)	10 (33.3)	8 (26.7)	9 (30)	2.77	1.01	−0.147	−1.12
	Resolution	2 (6.7)	9 (30)	11 (36.7)	8 (26.7)	2.83	0.913	−0.232	−0.786
	Class time	2 (6.7)	8 (26.7)	9 (30)	11 (36.7)	2.97	0.964	−0.424	−0.912
	Ratings ^a^	4 (13.3)	7 (23.3)	9 (30)	10 (33.3)	2.83	1.05	−0.404	−1.02
	Teacher-ratings ^a^	5 (16.7)	5 (16.7)	8 (26.7)	12 (40)	2.90	1.12	−0.570	−1.05

^a^ Established grade group (none: 1–4.9; few: 5–5.9; enough: 6–8.9; completely: 9–10).

**Table 5:** Study of the value of independence between CGP_1,_ CGS_2,_ EGP_1,_ EGS_2_.

	Variables	µ (X1–X2)	*t_n1+n2-2_*	df	*d*	r_xy_
**Primary Education (*n* = 55)**	Motivation	−0.378 (2.21–2.59)	n.s.	53	−0.035	0.209
	Teacher–student	−0.591 (1.96–2.56)	−2.416 *	53	0.000	0.315
	Student–content	−0.368 (1.93–2.30)	n.s.	53	0.030	0.217
	Student–student	−0.192 (2.18–2.37)	n.s.	53	0.018	0.106
	Autonomy	−0.950 (1.64–2.59)	−3.816 **	53	0.279	0.465
	Collaboration	−0.340 (2.18–2.52)	n.s	53	0.087	0.171
	Deepening	−0.981 (1.50–2.48)	−3.733 **	53	0.186	0.459
	Resolution	−0.443 (1.96–2.41)	n.s	53	0.028	0.223
	Class time	−0.991 (1.75–2.74)	−3.560 **	53	0.178	0.439
	Ratings ^a^	−0.626 (1.89–2.52)	−2.402 *	53	0.180	0.313
	Teacher-ratings ^a^	−0.597 (2.11–2.70)	−2.185 *	53	0.130	0.287
**Secondary Education (*n* = 60)**	Motivation	−0.500 (2.20–2.70)	−2.063 *	58	0.079	0.261
	Teacher–student	−0.600 (1.93–2.53)	−2.423 *	58	−0.024	0.303
	Student–content	−0.733 (1.83–2.57)	−3.440 **	58	0.136	0.412
	Student–student	−0.400 (2.20−2.60)	n.s	58	−0.080	0.193
	Autonomy	−1.00 (1.80–2.80)	−4.573 **	58	0.073	0.515
	Collaboration	−0.900 (2.03–2.93)	−3.521 **	58	0.045	0.420
	Deepening	−1.00 (1.77–2.77)	−4.225 **	58	0.098	0.485
	Resolution	−0.933 (1.90–2.83)	−3.938 **	58	0.093	0.459
	Class time	−1.36 (1.60–2.97)	−6.208 **	58	0.143	0.632
	Ratings ^a^	−1.00 (1.83–2.83)	−3.930 **	58	0.059	0.459
	Teacher-ratings ^a^	−0.867 (2.03–2.90)	−3.063 **	58	0.034	0.373

** The correlation is significant at the 0.01 level; * the correlation is significant at the 0.05 level; n.s. not significant; ^a^ established grade group (none: 1–4.9; few: 5–5.9; enough: 6–8.9; completely: 9–10).
